# DBP-iDWT: Improving DNA-Binding Proteins Prediction Using Multi-Perspective Evolutionary Profile and Discrete Wavelet Transform

**DOI:** 10.1155/2022/2987407

**Published:** 2022-09-28

**Authors:** Farman Ali, Omar Barukab, Ajay B Gadicha, Shruti Patil, Omar Alghushairy, Akram Y. Sarhan

**Affiliations:** ^1^Department of Elementary and Secondary Education, Peshawar, Khyber Pakhtunkhwa, Pakistan; ^2^Faculty of Computing and Information Technology, King Abdulaziz University, Rabigh, Jeddah 21911, Saudi Arabia; ^3^Department of Computer Science and Engineering, P.R. Pote, Collage of Engineering and Management, Amravati, India; ^4^Symbiosis Institute of Technology, Symbiosis Centre for Applied Artificial Intelligence, Symbiosis International University, Pune, India; ^5^Department of Information Systems and Technology, College of Computer Science and Engineering, University of Jeddah, Jeddah, Saudi Arabia; ^6^Department of Information Technology, College of Computing and Information Technology at Khulais, University of Jeddah, Jeddah, Saudi Arabia

## Abstract

DNA-binding proteins (DBPs) have crucial biotic activities including DNA replication, recombination, and transcription. DBPs are highly concerned with chronic diseases and are used in the manufacturing of antibiotics and steroids. A series of predictors were established to identify DBPs. However, researchers are still working to further enhance the identification of DBPs. This research designed a novel predictor to identify DBPs more accurately. The features from the sequences are transformed by F-PSSM (Filtered position-specific scoring matrix), PSSM-DPC (Position specific scoring matrix-dipeptide composition), and R-PSSM (Reduced position-specific scoring matrix). To eliminate the noisy attributes, we extended DWT (discrete wavelet transform) to F-PSSM, PSSM-DPC, and R-PSSM and introduced three novel descriptors, namely, F-PSSM-DWT, PSSM-DPC-DWT, and R-PSSM-DWT. Onward, the training of the four models were performed using LiXGB (Light eXtreme gradient boosting), XGB (eXtreme gradient boosting, ERT (extremely randomized trees), and Adaboost. LiXGB with R-PSSM-DWT has attained 6.55% higher accuracy on training and 5.93% on testing dataset than the best existing predictors. The results reveal the excellent performance of our novel predictor over the past studies. DBP-iDWT would be fruitful for establishing more operative therapeutic strategies for fatal disease treatment.

## 1. Introduction

DNA-binding proteins perform many crucial activities like DNA translation, repair, translation, and damage [1]. DBPs are directly encoded into the genome of about 2–5% of the prokaryotic and 6–7% of eukaryotic [2]. Several DBPs are responsible for gene transcription and replication, and some DBPs shape the DNA into a specific structure, called chromatin [3]. The research on DBPs is significant in diverse fatal disease treatment and production of drugs. For instance, nuclear receptors are the key components of tamoxifen and bicalutamide medicines which are used in cancer treatment. Similarly, glucocorticoid receptors participate in the production of dexamethasone, which is utilized in autoimmune diseases and anti-inflammatory, allergies, and asthma treatment [4–6]. Onward, Inhibitor of DNA-binding (ID) proteins are closely related to tumor-associated processes including chemoresistance, tumorigenesis, and angiogenesis. In addition, ID proteins are also directly concerned with lung, cervical, and prostate cancers [7].

Protein sequences are rapidly growing in the online database. A series of predictors were developed for diverse biological problems including iRNA-PseTNC [8], iACP-GAEnsC [9], cACP-2LFS [10], DP-BINDER [11], Deep-AntiFP [12], cACP [13], iAtbP-Hyb-EnC [14], iAFPs-EnC-GA [15], and cACP-DeepGram [16]. It is highly demanding to predict DBPs by computational approaches. Several predictors were introduced using the primary sequential information and structural features. Structured-based predictors produce good prediction results, but structural features for all proteins are unavailable. Some of the structure-based protocols are iDBPs [17], DBD-Hunter [18], and Seq(DNA) [19]. Sequence-based systems have been developed using sequential information, more convenient and easy to employ for large datasets. Therefore, many sequence-based systems were adopted for DNA-binding proteins identification. Among these methods: DBP-DeepCNN [20], DNA-Prot [21], iDNA-Prot [22], iDNA-Prot|dis [23], Kmer1 + ACC [24], Local-DPP [25], DBPPred-PDSD [26], DPP-PseAAC [27], and StackDPPred [28]. Consequently, Li et al. extracted features by a convolutional neural network (CNN) and Bi-LSTM [29]. Onward, Zhao et al. the features of the proteins are analyzed by six methods and classification is performed with XGBoost [30]. Each computational method contributed well to enhancing the prediction of DBPs. However, more efforts are needed to improve prediction of DBPs. Considering this, a new method (DBP-iDWT) is established to identify DBPs accurately. The contribution of our research is as follows:

- Designed three new feature descriptors i.e., F-PSSM-DWT, PSSM-DPC-DWT, and R-PSSM-DWT
- LiXGB is applied for model training and prediction
- Constructed a new computational model (DBP-iDWT) for improving DBPs identification

**Figure 1:**
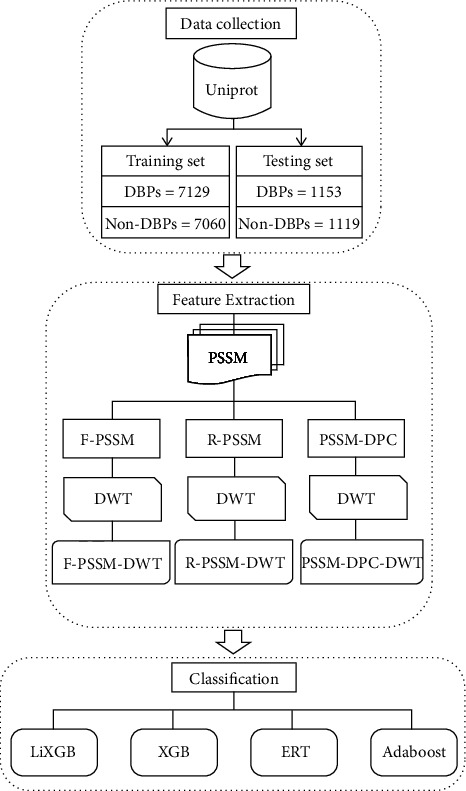
Architecture of the proposed model.

In addition to LiXGB, the features set is fed into three classification algorithms, namely ERT, XGB, and Adaboost. The efficacy of each classifier was assessed with ten-fold test, while the generalization capability was assessed by a testing set. LiXGB using R-PSSM-DWT secured the highest prediction outcomes than past methods. The flowchart of the DBP-iDWT is depicted in Figure 1.

The rest portion of the manuscript comprises three parts.  Section 2 comprises details regarding datasets and methodologies; in Section 3, the performance of classifiers has illustrated; and Section 4 summarizes the conclusion.

## 2. Materials and Methods

### 2.1. Selection of Datasets

We selected two datasets from the previous work [31]. One dataset (PDB14189) is employed model training and the other dataset is deployed as a testing dataset. PDB14189 was collected from the UniProt database [32]. To design a standard dataset, they removed more than 25% of similar sequences by CD-HIT toolkit. The final training dataset comprises 7129 DBPs and 7060 non-DBPs. The independent set was retrieved by a procedure explained in reference [33]. The similar sequences with a cutoff value 25% are removed. The final testing dataset contains 1153 DBPs and 1119 non-DBPs.

### 2.2. Feature Descriptors

In this work, the patterns are discovered with PSSM-DPC-DWT, F-PSSM-DWT, and R-PSSM-DWT. These approaches are elaborated in the following parts.

#### 2.2.1. Position-specific Scoring Matrix (PSSM)

Recently, evolutionary features are successfully implemented and improve the prediction results of many predictors [1, 20]. We also implemented PSSM for the formulation of evolutionary patterns. Each sequence is searched against the NCBI database applying the PSI-BLAST program for the alignment of homologous features [34].

The PSSM can be denoted as follows:

(1) $$\begin{array}{c} \begin{array}{c} PSSM={\left(P_{1},P_{2},\ldots ,P_{j},\ldots ,P_{20}\right)}^{T},\\ P_{i,j}=\left(P_{1,j},P_{2,j},\ldots ,P_{L,j}\right),\left(i=1,2,\ldots ,L\right), \end{array} \end{array}$$

where *T*and *P*_*i*,*j*_ indicate the transpose operator and score of *j* type of amino acid in the *i*^th^ position of query sequence.

#### 2.2.2. Filtered Position-specific Scoring Matrix (F-PSSM)

PSSM transforms the evolutionary patterns into numerical forms. It may comprise some negative scores which can lead to generating similar feature vectors despite different sequences. To cope with this hurdle, F-PSSM filters all the negative scores in the preprocessing step. The detail of dimension formulation is provided in [35].

#### 2.2.3. Position-specific Scoring Matrix-Dipeptide Composition (PSSM-DPC)

The local sequence-order patterns contains informative feature which are explored by incorporating DPC into PSSM. DPC calculates the frequency of continuous amino acids and produces a dimension of 400 [36]. DPC is calculated as follows:

(2) $$\begin{array}{c} PSSM-DPC={\left(G_{1,1},{\ldots ,G}_{1,20},G_{2,1},\ldots ,G_{2,20},\ldots ,G_{20,1},\ldots ,G_{20,20}\right)}^{T}\text{,} \end{array}$$

where

(3) $$\begin{array}{c} P_{i,j}=\frac{1}{L}\overset{L-1}{\underset{k=1}{\sum }}G_{k,i}\times G_{k+1,j}\left(1\leq i,j\leq 20\right)\text{.} \end{array}$$

#### 2.2.4. Reduced Position Specific Scoring Matrix (R-PSSM)

It is believed that there exist several similarities among 20 unique amino acids. Based on these similarities, researchers categorized these residues into groups. Li et al. [37] suggested that according to some specific residue the following groups can be formed:

(4) $$\begin{array}{c} G\left(i\right)=\left\{\begin{array}{cc} Y\text{,} & ifi=F,Y,W\text{;}\\ L\text{,} & ifi=M,L\text{;}\\ V\text{,} & ifi=I,V\text{;}\\ S\text{,} & ifi=A,T,S\text{;}\\ N\text{,} & ifi=N,H\text{;}\\ E\text{,} & ifi=Q,E,D\text{;}\\ K\text{,} & ifi=R,K\text{;}\\ i\text{,} & otherwise. \end{array}\right. \end{array}$$

Using the Li et al. rule, the *L* × 20 PSSM is converted to *L* × 10 matrix by the following equations:

(5) $$\begin{array}{c} G_{1}=\frac{F+Y+W}{3}\text{,}\\ G_{2}=\frac{M+L}{2}\text{,}\\ G_{3}=\frac{I+V}{2}\text{,}\\ G_{4}=\frac{A+T+S}{3}\text{,}\\ G_{5}=\frac{N+H}{2}\text{,}\\ G_{6}=\frac{Q+E+D}{3}\text{,}\\ G_{7}=\frac{R+K}{2}\text{,}\\ G_{8}=C\text{,}\\ G_{9}=G\text{,}\\ G_{10}=P\text{.} \end{array}$$

If *r*_1_*r*_2_*r*_3_ … ….*r*_*L*_ is a given protein sequence, then its reduced PSSM (R-PSSM) is indicated as follows:

(6) $$\begin{array}{c} RP=\left[\begin{array}{ccccccccccc} 1 & 2 & 3 & 4 & 5 & 6 & 7 & 8 & 9 & 10 & \;\\ r_{1} & R_{1,1} & R_{1,2} & R_{1,3} & R_{1,4} & R_{1,5} & R_{1,6} & R_{1,7} & R_{1,8} & R_{1,9} & R_{1,10}\\ r_{2} & R_{2,1} & R_{2,2} & R_{2,3} & R_{2,4} & R_{2,5} & R_{2,6} & R_{2,7} & R_{2,8} & R_{2,9} & \begin{array}{c} R_{2,10} \end{array}\\ \ldots & \ldots & \ldots & \; & \; & \; & \; & \; & \; & \; & \;\\ r_{L} & R_{L,1} & R_{L,2} & R_{L,3} & R_{L,4} & R_{L,5} & R_{L,6} & R_{L,7} & R_{L,8} & R_{L,9} & \begin{array}{c} R_{L,10} \end{array} \end{array}\right]\text{.} \end{array}$$

We obtain 110 feature vector from RP.

#### 2.2.5. Discrete Wavelet Transform

To achieve only salient information, some compression approaches like DWT is applied in research areas. DWT is used for compression of signals and denoising [38, 39]. DWT divides a signal into low-frequency and high-frequency components [40]. Low frequencies are more important than high-frequencies [41]. The Low frequencies are onward split into low and high levels to achieve discriminative patterns. DWT is computed as follows:

(7) $$\begin{array}{c} X\left(m,n\right)=\sqrt{\frac{1}{m}}\;\overset{y}{\underset{0}{\int }}f\left(y\right)\Psi \left(\frac{y-n}{m}\right)d_{y}\text{,} \end{array}$$

where *m*represents the scale variable and n shows the translation variable. *X*(*m*, *n*) is the transform coefficient. The low and high frequencies of a signal *f(t)* is computed as follows:

(8) $$\begin{array}{c} \begin{array}{c} C_{i,low}\left[a\right]=\overset{N}{\underset{k=1}{\sum }}s\left[k\right]L\left[2a-k\right],\\ C_{i,high}\left[a\right]=\overset{N}{\underset{k=1}{\sum }}s\left[k\right]H\left[2a-k\right], \end{array} \end{array}$$

where *C*_*i*,*high*_[*a*] and *C*_*i*,*low*_[*a*] are the high and low frequencies of the signal. *H*, *s[k]*, and *L,* represent the high pass filter, discrete signal, and low pass filter, respectively.

**Figure 2:**
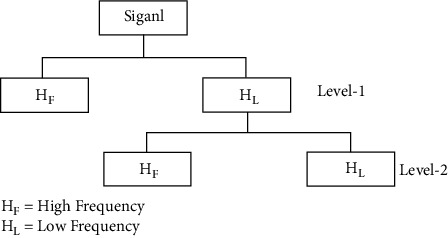
2-level structure of DWT.

To obtain only important features and eliminate the less informative and noisy patterns, DWT is extended into F-PSSM, PSSM-DPC, and R-PSSM to split into low and high frequencies up to two levels. Finally, PSSM-DPC-DWT, R-PSSM-DWT, and F-PSSM-DWT novel feature descriptors are constructed. The dimension of each feature set is 512 after applying DWT.  Figure 2 depicts the schematic view of Two-level DWT.

### 2.3. Light eXtreme Gradient Boosting

During the establishment of the predictor, the model training is performed by a classifier. Gradient Boosting Machine (GBM) classifier uses decision trees for the construction of a model. The model performance is improved with loss function [42]. Unlike GBM, eXtreme Gradient Boosting (XGB) employs an objective function. XGB concatenates loss function and regularization for regulating the model complexity. It performs parallel computations to optimize the computational speed. Due to these benefits of XGB, Light eXtreme Gradient Boosting (LiXGB) was proposed [43]. LiXGB possesses many additional features like lower memory, higher efficiency, and fast model training speed that improve the model performance. LiXGB minimizes the model training time of the large datasets. We utilized the hyperparameters like max depth, estimator, eta, lambda, and alpha. The “eta” maintains the learning rate, “estimator” constructs trees, “max depth” is used for controlling the tree depth, “alpha” shrinks the high dimension of the dataset, and “lambda” avoids the overfitting. Other parameters have been kept as default. These hyperparameters are also summarized in Table 1.

### 2.4. Proposed Model Validation Methodologies

The model performance is examined by different validation approaches The commonly used validation methods are k-fold and jackknife [44–47]. However, the jackknife is time-consuming and costly [48–50]. During 10-fold cross validation, training set is split into 10-folds. The 9 folds are used for model training and 1 fold is used for model validation. This process is repeated 10 times so that each fold is used for the test exactly once. The final prediction is the average of all tested folds [51–54]. The current work performance is evaluated with 10-fold and five indexes, i.e., specificity (Sp), F-measure, sensitivity (Sn), accuracy (Acc), and Mathew's correlation coefficient (MCC) for evaluating the model performance [55–58]. These parameters are computed as follows:

(9) $$\begin{array}{c} Acc=1-\frac{H_{-}^{+}+H_{+}^{-}}{H^{+}+H^{-}}\text{,}\\ Sn=1-\frac{H_{-}^{+}}{H^{+}}\text{,}\\ Sp=1-\frac{H_{+}^{-}}{H^{-}}\text{,}\\ MCC=\frac{1-\left(H_{-}^{+}+H_{+}^{-}/H^{+}+H^{-}\right)}{\sqrt{\left(1+\left(H_{-}^{+}+H_{+}^{-}/H^{+}\right)\right)\left(1+\left(H_{-}^{+}+H_{+}^{-}/H^{-}\right)\right)}}\text{,}\\ F1\;Score=\frac{\left(2*precision*recall\right)}{\left(precision+recall\right)}\text{,}\\ Precision=\frac{H^{+}}{H_{-}^{+}+H^{+}}\text{,}\\ Recall=\frac{H^{+}}{H_{+}^{-}+H^{+}}\text{,} \end{array}$$

where *H*^+^ is used to denote the DBPs, *H*^−^ is the non-DBPs, *H*_+_^−^ shows the prediction of non-DBPs which the model predicted mistakenly as DBPs, and *H*_−_^+^ represents the DBPs which are classified by the model as non-DBPs.

## 3. Results and Discussion

After performing experiments on the models, In this part, we will elaborate the obtained results of the learning algorithms via the extracted feature sets of the training and testing sequences.

### 3.1. Results of Feature Encoders before DWT

In this section, we have reported the outcomes of F-PSSM, PSSM-DPC, and R-PSSM in Table 2. The performance of the individual descriptor is analyzed by 10-fold test and assessment indices. On F-PSSM, the accuracies secured by LiXGB, XGB, ERT, and Adaboost are 76.60%, 74.57%, 75.18%, and 71.52%, respectively. Among all classifiers, LiXGB achieved the best accuracy. On PSSM-DPC, all classifiers enhanced the prediction results and generated 83.62%, 81.63%, 79.56%, and 80.07% accuracies by LiXGB, XGB, ERT, and Adaboost, respectively. Similarly, the classifiers also improved the performance on the R-PSSM descriptor using all evaluation parameters. LiXGB attained the highest (83.62%) accuracy. The predictions indicate that LiXGB possesses higher learning power comparatively XGB, ERT, and Adaboost.

### 3.2. Results of Feature Encoders after DWT

The features extracted by representative methods may contain some noisy, redundant, or less informative features. To avoid such features, DWT is applied to F-PSSM, PSSM-DPC, and R-PSSM. DWT considers the informative patterns and improves the performance of the model. After applying DWT, we achieve F-PSSM-DWT, PSSM-DPC-DWT, and R-PSSM-DWT. Each feature is fed into Adaboost, ERT, XGB, and LiXGB in order to examine the performance over these feature descriptors and results are summarized in Table 3. With 10-fold test, Adaboost, ERT, XGB, and LiXGB produced 73.20%, 77.26%, 75.37%, and 79.40% accuracies which are 1.68%, 2.08%, 0.80%, and 2.80% than F-PSSM, PSSM-DPC, and R-PSSM, respectively. Similarly, the classifiers also boosted the performance on PSSM-DPC-DWT on all evaluation parameters. Furthermore, with R-PSSM-DWT, Adaboost, ERT, XGB, and LiXGB have enhanced the accuracies by 2.16%, 3.49%, 1.98%, and 3.22% than R-PSSM. These results demonstrate that all classifiers show improvement in performance after applying DWT. Among all feature descriptors, the best results are secured by R-PSSM-DWT.

LiXGB has constantly depicted better achievement than other classifiers. LiXGB enhanced the performance and generated 3.23%, 3.79%, and 4.61% higher accuracies than XGB, ERT, and Adaboost with R-PSSM-DWT. It is concluded that the performance of LiXGB is superior to other classifiers.

### 3.3. Comparison with Existing Predictors Using Training Set

Several methods have been implemented for the identification of DBPs. The proposed work is compared with past studies including iDNA-Prot [22], iDNA-Prot|dis [23], TargetDBP [59], MsDBP [60], PDBP-CNN [29], and XGBoost [30] and summarized the results in Table 4. Our proposed study improved the accuracy by 4.82%, sensitivity by 10.58%, and MCC by 0.09 than the best predictor (PDBP-CNN). Similarly, The DBP-iDWT enhanced 5.42% Acc, 2.49% Sn, 8.65% Sp, and 0.11 MCC than the second best study (XGBoost). In the same fashion, our predictor performance is superior to past studies using all four assessment parameters. The outcomes verified that DBP-iDWT can discriminate DBPs with high precision.

### 3.4. Comparison with Past Predictors Using Independent Set

A method is considered effective if it has high generality for the new sequences. We also evaluated the proposed work using a testing dataset. The results compared with past studies like PseDNA-Pro, iDNAPro-PseAAC, iDNAProt-ES, DPP-PseAAC, TargetDBP, MsDBP, and PDBP-Fusion as noted in Table 5. It is noted that our predictor (DBP-iDWT) raised 5.06% Acc, 17.06% Sn, 8.22% Sp, and 0.10 MCC than PDBP-Fusion. Similarly, DBP-iDWT improved 6.14% Acc, 14.02% Sn, and 0.13 MCC than TargetDBP. Onward, the proposed study also secured higher prediction results than other past methods in Table 5.

These results analysis confirm that the incorporation of DWT into R-PSSM in conjunction with LiXGB can identify DBPs more accurately. Past studies have reported that the selection of the best features can improve the model performance [61–63]. In this study, we also implemented feature selection approach including mRmR and SVM-RFE, however, no improvement in the model performance is observed.

## 4. Conclusion and Future Vision

DBPs play an active role in many biological functions and drug designing. We have designed a predictor for improving DBPs prediction with high precision. The global information, local features, sequence-order patterns, and correlated factors are explored by PSSM-DPC-DWT, R-PSSM-DWT, and PSSM-DPC-DWT.

The models are trained with LiXGB, XGB, ERT, and Adaboost. It is concluded that R-PSSM-DWT with LiXGB has effectively attained superlative performance than other predictors. The successful outcomes of the proposed study is due to factors like utilization of effective descriptors, application of a compression scheme, and appropriate classifier.

DBP-iDWT will be effective for the identification of DBPs due to its promising prediction power than other predictors and perform an active role in drug development. DBP-iDWT would be fruitful for establishing more operative therapeutic strategies for fatal disease treatment. In addition, we will apply advanced deep learning frameworks [64–67] in our future work to further improve the DBPs prediction.

## Figures and Tables

**Table 1:** Applied parameters with values.

Parameter	Value
Era	0.1
No. of estimator	500
Alpha	1
Lambda	1
Max depth	8

**Table 2:** Results of encoders before DWT.

Model	Encoder	Acc (%)	Sn (%)	Sp (%)	MCC (%)
Adaboost	F-PSSM	71.52	80.42	62.54	43.67
	PSSM-DPC	80.05	78.44	81.69	60.15
	R-PSSM	80.07	76.15	84.02	60.35

ERT	F-PSSM	75.18	84.74	58.97	44.56
	PSSM-DPC	79.22	73.18	85.31	58.42
	R-PSSM	79.56	74.99	84.18	59.40

XGB	F-PSSM	74.57	82.17	66.90	49.67
	PSSM-DPC	81.53	76.15	86.97	63.47
	R-PSSM	81.63	76.48	86.84	63.64

LiXGB	F-PSSM	76.60	82.47	66.01	48.75
	PSSM-DPC	83.54	84.61	82.46	67.10
	R-PSSM	83.62	82.30	84.96	67.27

**Table 3:** Results of feature encoders after DWT.

Model	Encoder	Acc (%)	Sn (%)	Sp (%)	MCC (%)
Adaboost	F-PSSM-DWT	73.20	82.35	56.67	40.21
	PSSM-DPC-DWT	81.81	80.45	83.19	63.66
	R-PSSM-DWT	82.23	77.68	86.81	64.77

ERT	F-PSSM-DWT	77.26	79.91	74.59	54.58
	PSSM-DPC-DWT	81.53	76.15	86.97	63.47
	R-PSSM-DWT	83.05	81.30	84.82	66.15

XGB	F-PSSM-DWT	75.37	83.43	60.81	45.31
	PSSM-DPC-DWT	82.45	83.65	81.25	64.91
	R-PSSM-DWT	83.61	82.66	84.56	67.23

LiXGB	F-PSSM-DWT	79.40	83.11	75.65	58.94
	PSSM-DPC-DWT	84.74	84.30	85.19	69.49
	R-PSSM-DWT	86.84	86.60	87.08	73.69

**Table 4:** Comparative analysis with past work on the training set.

Predictor	Acc (%)	Sn (%)	Sp (%)	MCC
iDNA-prot	75.40	83.81	64.73	0.50
iDNA-prot|dis	77.30	79.40	75.27	0.54
TargetDBP	79.71	79.56	79.85	0.59
MsDBP	80.29	80.87	79.72	0.60
PDBP-CNN	82.02	87.49	76.50	0.64
XGBoost	81.42	84.11	78.43	0.62
DBP-iDWT	86.84	86.60	87.08	0.73

**Table 5:** Comparative analysis with past work using testing dataset.

Predictor	Acc (%)	Sn (%)	Sp (%)	MCC
PseDNA-pro	67.23	78.38	56.08	0.35
iDNAPro-PseAAC	66.22	78.37	54.05	0.33
iDNAProt-ES	68.58	95.95	41.22	0.44
DPP-PseAAC	61.15	55.41	66.89	0.22
TargetDBP	76.69	76.35	77.03	0.53
MsDBP	66.99	70.69	63.18	0.33
PDBP-fusion	77.77	73.31	66.85	0.56
DBP-iDWT	82.83	90.37	75.07	0.66

## Data Availability

The data and code are freely available at https://github.com/Farman335/DBP-DWTPred.
